# Supplementary material for: Enhanced Luminescent Detection of Circulating Tumor Cells by a 3D Printed Immunomagnetic Concentrator
Source: Biosensors (Basel). 2021 Aug 17;11(8):278. doi: 10.3390/bios11080278 (PMC8391490; doi:10.3390/bios11080278)
Supplement: Supplementary file 1 [file biosensors-11-00278-s001.zip › biosensors-1330603-supplementary.pdf]

biosensors

*Supplementary Materials*

# Enhanced Luminescent Detection of Circulating Tumor Cells by a 3D Printed Immunomagnetic Concentrator

**Chanyong Park [1,†], Abdurhaman Teyib Abafogi [2,†], Dinesh Veeran Ponnuvelu [2], Ilchan Song [3], Kisung Ko [3] and Sungsu Park [2,4,5,*]**

1 Department of Medical Device, Korea Institute of Machinery & Materials (KIMM), Daegu 42994, Korea; cksdyd6348@skku.edu
2 School of Mechanical Engineering, Sungkyunkwan University (SKKU), Suwon 16419, Korea; abditeyf@skku.edu (A.T.A.); dinesh@skku.edu (D.V.P.)
3 Department of Medicine, College of Medicine, Chung-Ang University, Seoul 06974, Korea; icsong82@cau.ac.kr (I.S.); ksko@cau.ac.kr (K.K.)
4 Department of Biomedical Engineering, Sungkyunkwan University (SKKU), Suwon 16419, Korea
5 Institute of Quantum Biophysics (IQB), Sungkyunkwan University (SKKU), Suwon 16419, Korea
* Correspondence: nanopark@skku.edu; Tel.: +82-31-290-7431; Fax: +82-31-290-5889
† The authors contributed equally to this work.

**Citation:** Park, C.; Abafogi, A.T.; Ponnuvelu, D.V.; Song, I.; Ko, K.; Park, S. Enhanced Luminescent Detection of Circulating Tumor Cells by a 3D Printed Immunomagnetic Concentrator. *Biosensors* **2021**, *11*, 278. https://doi.org/10.3390/bios11080278

Received: 27 July 2021
Accepted: 16 August 2021
Published: 17 August 2021

**Publisher's Note:** MDPI stays neutral with regard to jurisdictional claims in published maps and institutional affiliations.

**Copyright:** © 2021 by the authors. Submitted for possible open access publication under the terms and conditions of the Creative Commons Attribution (CC BY) license (http://creativecommons.org/licenses/by/4.0/).

## Surface plasmon resonance analysis

To compare the binding affinities of mAb (monoclonal antibody) CO17-1A and Anti-EpCAM mAb. Surface plasmon resonance (SPR) analyses were performed using a ProteOn XPR36 instrument (Bio-Rad, Hercules, CA). The EpCAM antigen (R&D systems, Minneapolis, MN) was immobilized onto the surface of a GLC sensor chip and stabilized with PBS-T buffer (PBS buffer containing 0.05% v/v Tween-20). After stabilization, anti-EpCAM mAb (600 nM) and mAbP COK (600 nM) were applied to the sensor chip with a flow rate of 80 μl/min at 25˚C. The surface of the GLC chip was regenerated using phosphoric acid. Data analyses were performed with ProteOn Manager 2.1 software, and data were corrected by subtraction of the zero-antibody concentration column as well as inter-spot correction.

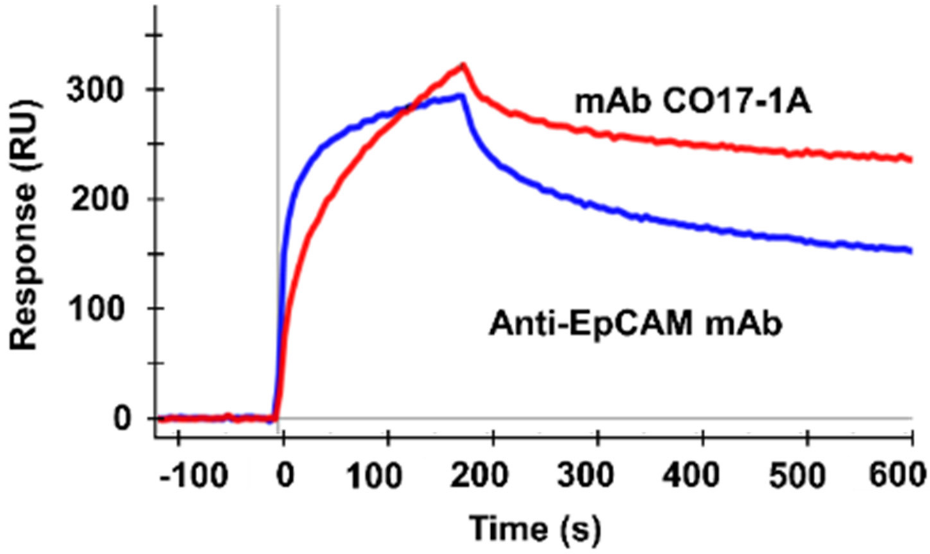


**Figure S1.** Kinetic analysis of antigen-antibody interaction on a sensor chip using Surface plasmon resonance (SPR). SPR analysis was used to confirm the binding activity of mAb CO17-1A, Anti-

EpCAM mAb to an epithelial cell adhesion molecule (EpCAM-Fc). To confirm the antigen-antibody binding activity of mAb CO17-1A purified from transgenic Arabidopsis plants, the surface of a GLC sensor chip was immobilized with EpCAM fused to human IgG Fc fragment (EpCAM-Fc) molecules. Anti-EpCAM mAb (600 nM), and mAb CO17-1A (600 nM) were applied to the sensor chip with a flow rate of 80 μL/min at 25˚C, and the response curves shown in the figure were consequently obtained.

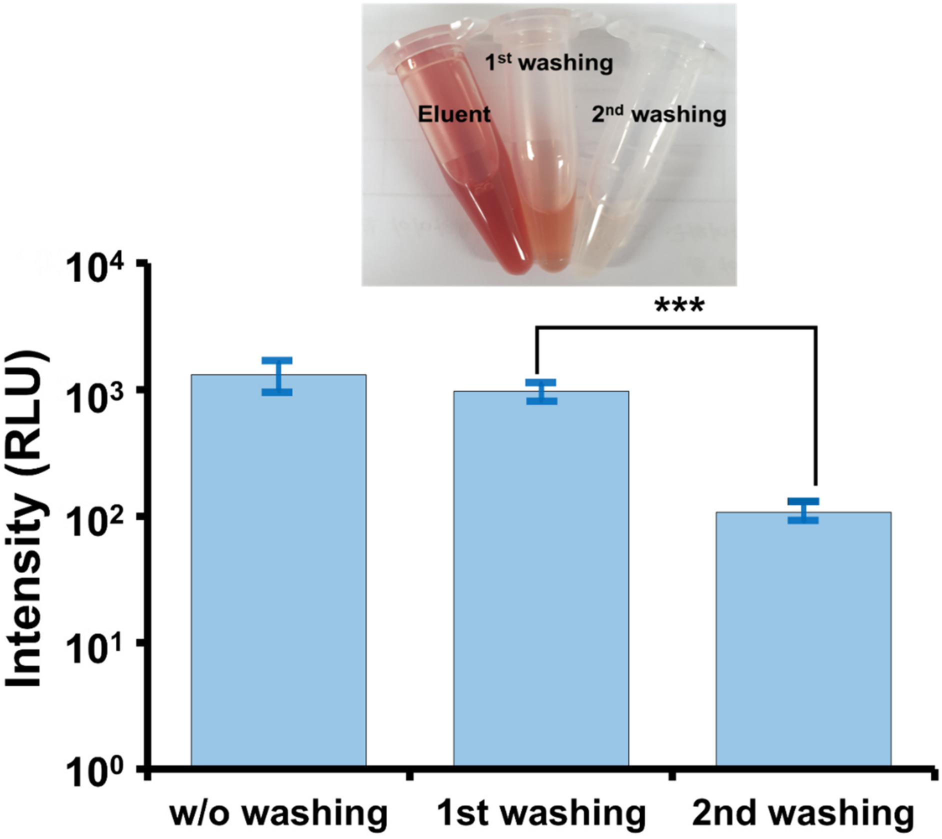


**Figure S2.** Effect of the washing step on the luminescence intensity. The blood is injected into 3DPIC and washed with PBS. Eluents were collected for each washing step, and the intensity was measured using ATP luminescence assay. Student t-test was used. ***: $p < 0.001$. Sample number (n) =3.
